# Real-Time *In Situ* Navigation System With Indocyanine Green Fluorescence for Sentinel Lymph Node Biopsy in Patients With Breast Cancer

**DOI:** 10.3389/fonc.2021.621914

**Published:** 2021-05-05

**Authors:** Zhaorui Wang, Xiaowei Yang, Jingjing Wang, Peng Liu, Yubo Pan, Chunguang Han, Jing Pei

**Affiliations:** ^1^ Department of General Surgery, First Affiliated Hospital of Anhui Medical University, Hefei, China; ^2^ Department of Precision Machinery and Precision Instrumentation, University of Science and Technology of China, Hefei, China

**Keywords:** breast cancer, sentinel (lymph) node biopsy, indocyanine green, real-time *in situ* navigation, false negative

## Abstract

**Background:**

The naked-eye invisibility of indocyanine green fluorescence limits the application of near-infrared fluorescence imaging (NIR) systems for real-time navigation during sentinel lymph node biopsy (SLNB) in patients with breast cancer undergoing surgery. This study aims to evaluate the effectiveness and safety of a novel NIR system in visualizing indocyanine green fluorescence images in the surgical field and the application value of combined methylene blue (MB) and the novel NIR system in SLNB.

**Methods:**

Sixty patients with clinical node-negative breast cancer received indocyanine green (ICG) and MB as tracers. Two NIR system instruments, namely, lymphatic fluorescence imaging system (LFIS) designed by the University of Science and Technology of China and vascular imager by Langfang Mingde Medical Biotechnology Co., Ltd. (Langfang vascular imager), were used as navigation assistance to locate sentinel lymph nodes (SLNs). Excising the lymph nodes developed by both MB and ICG by two NIR systems or palpably suspicious as SLNs and undergoing rapid pathological examination.

**Results:**

Both instruments exhibited 95% (57/60) success for real-time lymphatic fluorescent images. A total of 186 SLNs were identified, of which two were pathologically confirmed as lacking any lymph node tissue. SLN identification rate was 100% (184/184) for MB plus LFIS and 86.96% (160/184) for MB alone. The median number of SLNs identified by LFIS combined with MB was 3 (range of 1–8), which was significantly higher than that by MB alone at 2 (range 1–7) (P<0.05).

**Conclusion:**

LFIS effectively detects SLNs in breast cancer, projects the fluorescence signals during surgery, and provides a continuous surgical navigation system without the need for a remote monitor. The ICG method navigated by combined LFIS and MB may be a promising alternative tracer for radioisotope in SLN mapping.

**Clinical Trial Registration:**

This clinical trial was registered with the China Clinical Trial Center, registration number ChiCTR2000039542.

## Background

Sentinel lymph node biopsy (SLNB) is the standard treatment for clinical axillary lymph node-negative breast cancer (1). Clinical studies and meta-analysis showed that the exemption from axillary lymph node dissection for sentinel lymph node (SLN)-negative patients does not affect their overall survival and saves them from its complications, such as upper limb lymphedema, numbness, and pain (2). Radioisotope (RI) and blue dye are SLNB tracers that are globally used but have some limitations. Blue dye has a long learning curve, requires practice to achieve high accuracy, has the risk of anaphylactic reactions, and is widely used in China but has unsatisfactory detection rate. Meanwhile, RI has a high detection rate. Dual localization with both tracers is considered the standard method (3, 4) with high detection rate and low false negative rate of 5%–10% (5, 6). However, RI requires the assistance of nuclear medicine department, and its widespread use is limited by exposure to RIs and high preservation.

Indocyanine green (ICG) has been introduced as a new SLNB tracer since 2005 (7). The NIR system can visualize the lymphatic flow and provide navigation for the surgeon to find and remove the SLNs. ICG has higher detection rate than traditional tracers RI and blue dye (8, 9). Recent studies and meta-analysis indicated that ICG and RI tracers show no statistically significant difference in SLN detection rate (10, 11). Although ICG has a high detection rate and short learning curve, the conventional NIR system need to be improved. Surgeons need to look at the remote monitors to identify the location of the fluorescent image because the fluorescent signal is invisible to the naked eye. The shadowless operating lamp must also be turned off to decrease the white-light contamination of images. This study introduced a novel NIR system that provides real-time operation navigation by producing fluorescent images that are directly visible in the operation field. SLNB was assisted by two NIR systems to verify the feasibility and effectiveness of lymphatic fluorescence imaging system (LFIS). In addition, the fluorescence localization effectiveness of ICG combined with MB was evaluated.

## Material and Methods

### Study Design

This clinical trial was a single-arm, prospective, multicenter study. Participating surgeons were well trained for sentinel lymph node biopsy.

### Patients

Sixty female patients with early breast cancer and clinically confirmed negative axilla were recruited from the Department of Breast Surgery of the First Affiliated Hospital of Anhui Medical University of China Department of Tumor Surgery, The First Affiliated Hospital of Bengbu Medical College of China, and Department of Breast Surgery of Nantong Cancer Hospital of China between March 2018 and June 2018.

Inclusion criteria were as follows: 1) primary breast cancer confirmed by core needle biopsy or Mammotome biopsy; 2) no enlarged axillary lymph nodes as verified by palpation, mammography, or breast ultrasound examination; and 3) no distant metastasis.

Exclusion criteria were as follows: 1) pregnant or lactating, 2) primary breast cancer confirmed by open biopsy, 3) preoperative radiotherapy at the breast area or neoadjuvant chemotherapy, 4) history of the axillary surgery, and 5) allergy to iodine.

This study was approved by the Institutional Research Ethics Committee of the First Affiliated Hospital of Anhui Medical University, The First Affiliated Hospital of Bengbu Medical College of China and Nantong Cancer Hospital of China. And written informed consent was obtained from each subject. The study protocol was registered : (ChiCTR2000039542, Chinese Clinical Trial Registry).

### Imaging System and Reagents

**Figure 1:**
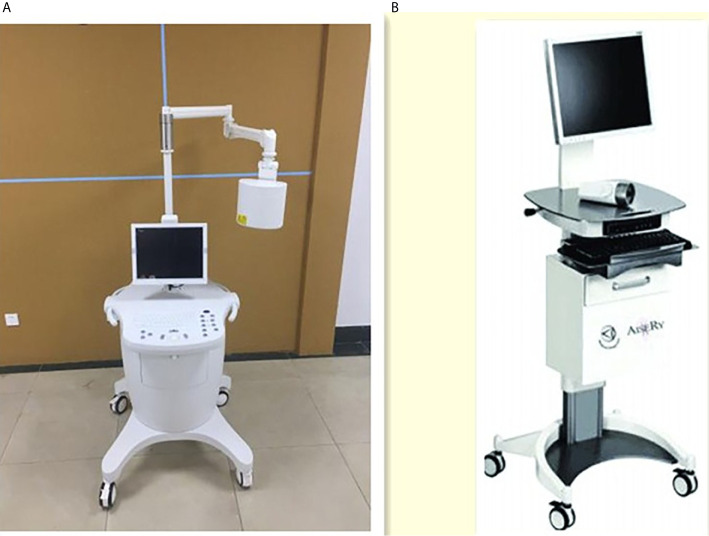
Two NIR system. **(A)** Left one: Lymphatic Fluorescence Imaging System(LFIS) designed by university of Science and Technology of China. **(B)** Right one: Vascular imager by Langfang Mingde Medical Biotechnology Co.LTD (Langfang Vascular imager).

The two kinds of NIR system used in this study were LFIS by the University of Science and Technology of China and Jiangsu Xinmei Medical Engineering Technology Co., Ltd. (**Figure 1A**) and vascular imager by Langfang Mingde Medical Biotechnology Co., Ltd. (Langfang vascular imager) (**Figure 1B**). The fluorescence emission from the surgical site is acquired by the LFIS device, calibrated based on the detected working distance, and projected back to the surgical site for surgical guidance. This instrument has the updated version of handheld projective imaging device (12). The differences between Langfang vascular imager and LFIS is that the LFIS projects fluorescent images onto the skin surface. Under the Langfang vascular imager navigation, surgeons need to look at the remote monitors to identify the location of the fluorescent image because the fluorescent signal is invisible to the naked eye.

The tracers used were MB (JUMPCAN PHARMACEUTICAL GROUP CO., LTG.) and ICG (Eisai, Liaoning Pharmaceutical Co., Ltd.).

Preparation of ICG solution : Indocyanine green dosage form is powder, each containing 25mg. Dissolve with sterilization water of 10ml originally prepared by the manufacturer, and the mass concentration after dissolution is 2.5mg/ml. Then 0.2 mL of 2.5mg/mL indocyanine green was diluted to 1 mL (0.5mg/mL) with sterilized injection water as the mass concentration used.

In the studies on the influence of ICG concentration on the number of lymph node detection and lymphatic vessel development, the concentration varied from 0.5mg/ml to 2.5mg/ml, but the development effect of lymphatic vessel and lymph node was ideal.

### SLNB Procedure

**Figure 2:**
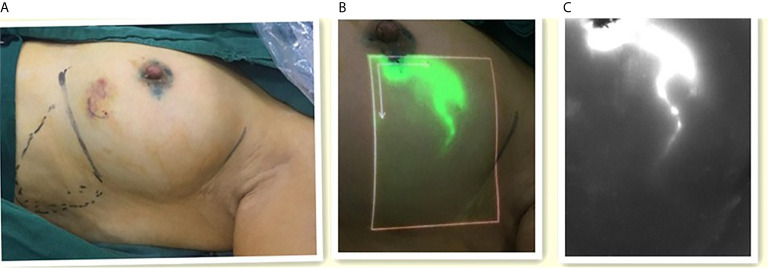
The images of lymphatic drainage. **(A)** Left one: ICG was injected around the areola. **(B)** Middle one: The image of lymphatic drainage on the skin with the LFIS. **(C)** Right one: The image of lymphatic drainage on the monitor with the Langfang Vascular imager.

After general anesthesia was administered, the four sites of periareolar region were subcutaneously injected with 1 ml of 1% MB (**Figure 2A**), followed by 1 ml of ICG after 5 minutes. The areola area was then massaged. Real-time imaging of lymphatic drainage imagine in the outer upper quadrant of the breast was performed using Langfang vascular imager and LFIS, and images of lymphatic drainage were captured (**Figures 2B, C**). SLNB incision was selected 2 cm away from where the fluorescence disappeared, and the consistency of incision location was evaluated. If the subcutaneous lymphatic flow is invisible or is discontinuous, then conventional incision (the external margin of the pectoralis major and the anterior margin of the latissimus dorsi) is chosen.

**Figure 3:**
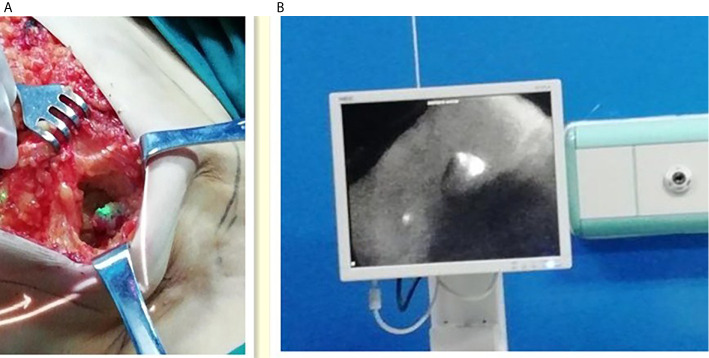
Intraoperative fluorescence imaging of lymph nodes. **(A)** Left one: Intraoperative fluorescence imaging of lymph nodes with the LFIS. **(B)** Right one: Intraoperative fluorescence imaging of lymph nodes with the Langfang Vascular imager.

**Figure 4:**
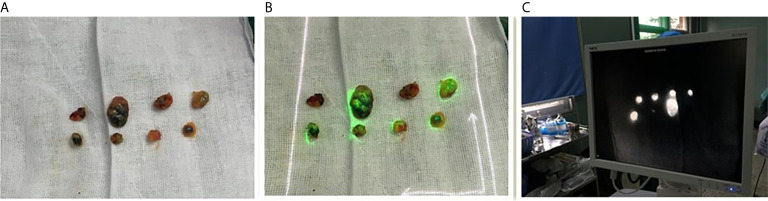
Image of resected lymph nodes. **(A)** Left one: The MB lymph node. **(B)** Middle one: The ICG lymph nodes with the LFIS. **(C)** Right one: The ICG lymph nodes with the Langfang Vascular imager.

The fluorescent (ICG-positive) (**Figure 3**) and/or blue (MB-positive) lymph nodes were localized and excised similarly to the SLNs. The axilla was inspected for residual fluorescent with the Langfang vascular imager or blue nodes. Lymph node development was recorded, particularly whether the lymph node is MB-, LFIS+, and Langfang+ (**Figure 4**). All excised nodes underwent immediate and postoperative pathological examinations. Axillary lymph node dissection was conducted only on patients with positive SLNs.

The overall research process is shown in **Image 1**.

### Statistical Analysis

SPSS statistical package version 21.0 was used for statistical analyses. Non-parametric Wilcoxon signed rank test was employed to compare the median number of SLNs between groups. A P-value<0.05 was considered statistically significant.

## Results

### Patient Characteristics

Patient and tumor characteristics are shown in **Table 1**.

**Table 1:** Tumor and patients characteristics.

Factors	N	%
Total patients	60	
Age, median (range)	50(31-67)	
Body Mass Index <24 ≥24 ≥28 ≥30	23.93(18.32-30.12) 31 22 6 1	51.67 36.66 10.00 1.67
Tumor stage Tis T1 T2 T3	6 24 28 2	10 40 46.67 3.33
Histological grade 1 2 3 Unknown	9 34 12 5	15 56.67 20 8.33
Patients with positive SLN	10	16.67

### ICG Mapping

Among the 60 patients with SLN detected using ICG combined with MB, real-time lymph-vessel fluorescent images were observed in the skin of 57 patients by both instruments in the same position. Hence, the rate of real-time observation of skin lymphatic streams was 95%. The three patients with no substantial lymphatic streams on the skin underwent SLNB with conventional incision, and their fluorescent lymph nodes were found successfully. In one case, 2 SLNs were ICG positive and MB negative, and pathology revealed no lymph node tissues.

With the exclusion of the above case, 184 SLNs were obtained. **Table 2** shows that the detection rate of LFIS combined with MB sentinel node was 100% (184/184), and that of MB alone was 86.96% (160/184). The median number of SLNs identified by LFIS combined with MB was significantly higher (3, range 1–8) than by MB (2, range 1–7) (P <0.05).

**Table 2:** Detection rate of various methods.

	Number of SLNs identified and detection rate	Number of patients identified detection rate
(MB+) and (LFIS+)	184 (100%)	60(100%)
Only MB+	160 (86.96%)	58(96.7%)
Only LFIS+	177 (96.20%)	60(100%)
Langfang Vascular imager	174 (94.57%)	60(100%)

Among the 60 patients, 10 has metastatic SLNs, and 15 out of 184 lymph nodes were positive. The trace situation of positive lymph nodes is shown in **Table 3**. The use of MB alone would have missed 20% of the positive axillae. Given the small number of cases, further clinical trials must be conducted for validation.

**Table 3:** Metastatic SLNs and patients with metastatic SLNs detected by different methods.

	Number of detected positive nodes and detection rate	Number of patients detected positive node and detection rate
LFIS(+) and MB(+)	12(80%)	8(80%)
LFIS(+) and MB(+)	3(2%)	2(20%)
LFIS(-) and MB(+)	0(0)	0(0)
Total	15(100%)	10(100%)

We analyzed the relationship between the number of sentinel lymph node biopsies and Body Mass Index (BMI). BMI was negatively correlated with the number of SLNs, and the correlation was weak.

### Adverse Effects

No allergic reaction was observed from surgery to discharge. All 60 patients were followed up for 24 months and showed no adverse reactions such as skin lesions, necrosis, and infection at the ICG injection site.

## Discussion

This prospective and self-matching study aimed to compare LFIS with conventional NIR system and blue dye method to assess whether the former can be used as a reliable alternative and whether it is superior over blue dye method for early breast cancer. The Langfang vascular imager has been approved for clinical use. The researchers took into account that the new system (LFIS) was in the clinical validation stage, and compared the consistent development rate of subcutaneous lymphatic vessels and the detection rate of lymph nodes with Langfang vascular imager. So the two NIR systems were used in my study.

ICG has a short half-life in plasma. After injection, it can bind tightly to plasma proteins and immediately enter lymphatic vessels to flow to SLNs (13). Thus, ICG serves as a marker for a specific molecule or cell. SLNs in breast cancer regularly occur in specific areas. Thus, the precise location of incision can be readily chosen, and SLNs are easy to find under fluorescence guidance. The NIR fluorescence band of ICG is 700–900 nm, which is invisible to the naked eye. Therefore, the position and movement of these molecules and cells must be assisted by a NIR fluorescence imaging system to obtain the accurate location of lymphatic vessels and sentinel lymph nodes. Although the ICG fluorescence method is unique in surgical navigation and has high sensitivity, its application to current NIR systems encounters several technical issues that must be addressed. Companies like Hamamatsu Photonics and Novadaq have their own near-infrared fluorescent navigation systems. The two companies’ instruments were similar to the Langfang vascular imager used in the study. This type of NIR system displays the fluorescence image on the mobile monitor, and the surgeon must alternately and repeatedly look at the surgical field and the remote monitor to confirm the site of the lymph nodes. With the fluorescence intensity image devoid of major anatomical land-marks, this phenomenon destroys the consistency and increases the complexity of the surgical procedure.

Our research team focuses on real-time in-situ surgical navigation. A Google-enhanced imaging system was developed in collaboration with the University of Science and Technology of China. When the surgeon wears Google Glasses, the fluorescent signal is projected onto the Google Glasses to achieve real-time display of approximate surgical field (14, 15). This study presents a novel NIR system called LFIS that can continuously and accurately project the fluorescence image on the surgical field to allow for a focused vision and shorten the operation time. The LFIS provides real-time navigation for SLNB and has two modes: projection mode and lighting mode, which shifts by pressing one button, thus limiting the need to switch the shadowless lamp. LFIS is comparable to conventional NIR systems in locating sentinel nodes. Owing to self-matching, quantitative comparison under short surgery duration is unavailable.

The detection rate of LFIS-guided ICG combined with MB (100%, 184/184) was better than that of MB alone (86.96% (160/184), and this finding was consistent with previous studies. The total number of LFIS positive (177) was higher than that of MB positive (160) and is possibly related to the affinity for the lymphatic system. The affinity of ICG is stronger than that of MB because of the molecular structure and diameter; the molecular mass of ICG (774.9) is larger than that of MB (319.9) (16).

ICG fluorescence imaging has been favored by researchers as a new SLN tracer method since 2005. Recent meta-analysis showed that ICG has SLN detection rate from 81.9% to 100% and sensitivity from 65.2% to 100%. No statistical difference in detection rate and sensitivity was found between ICG combined with RI tracer and ICG alone (17).

The key factor in evaluating the quality of SLNB is the false-negative rate. False negative means that metastatic lymph nodes are not detected, and the tumor stage is underestimated. This phenomenon leads to inadequate systemic treatment and increases the risk of local recurrence and distant metastasis. Another meta-analysis based on six studies reported 8% false negative rate when using ICG as a tracer (18). In the National Surgical Adjuvant Breast and Bowel Project B-32 trial including 5611 patients with clinically negative axillary lymph nodes, a false negative rate of 9.8% is found when using combined blue dye and RI double tracer (4); this value was comparable to that of ICG. Findings about the comparability of false negative rate (FNR) between ICG and dual tracer of RI and blue dye are inadequate and thus require additional clinical trials for validation. On the basis of the above data, MB combined with ICG can be a new dual-tracer method to replace RI plus blue dye, especially for institutions without access to RI. This novel method have the following advantages over the gold standard: a) projection real-time navigation with advanced image processing for lymphatic visualization, b) no involvement of physicians from the nuclear medicine department prior to the operation, and c) easy transportation and preservation.

The median number of removed SLNs is 1.5–3.4 under the guidance of conventional NIR system with ICG fluorescence (11) and 3 under guidance of individual LFIS and conventional NIR system. The median number of SLNs excised by blue dye is 2, which is significantly lower than that by ICG methods (P <0.05). Compared with blue dye, the NIR fluorescence imaging system shows sensitivity even at low concentrations that are visible to the naked eye (19) and detects more SLNs. The increase in the number of SLNs detected within the appropriate range can avoid excessive interference in the axillary tissue and enable an accurate and full evaluation of lymph node condition. Extracting only one SLN has a high risk of false negative. To date, 3–4 SLNs are needed to identify more than 97% positive lymph nodes. In combination with postoperative complications, the extraction of no less than 4 SLNs is currently recommended (20).

As for the result that ICG combined with MB is superior to MB alone, we believe that ICG and MB can complement each other. The ICG can show where the lymphatic vessels are going, and along the way we can find the sentinel lymph nodes, which have a navigation function. However, it is difficult to avoid the leakage of ICG after the removal of the first sentinel lymph node, resulting in the “pollution” of the fluorescent signal in the operative field, which increases the difficulty of subsequent lymph node biopsies. At this time, MB serves as a tracer for the naked eye to find blue-stained lymph nodes, providing further localization. In our study, 7 SLNs (3.8%) in the ICG + MB group were only blue, indicating that ICG alone may have resulted in partial lymph node escape.

In a case in this study, two SLNs were detected by the two fluorescence imaging systems but not by MB. These two lymph nodes were re moved and pathologically indicated as lacking lymph node tissues. This finding revealed the limitations of ICG as a tracer. The leakage of ICG caused by intraoperative lymphatic vessel damage and its high sensitivity resulted in the occurrence of non-lymph node fluorescence images in the operative field. This phenomenon is called “contamination”, which may cause the difficulty of lymph node localization.

In this study, three patients had no percutaneous fluorescence signal and superficial lymphangiography but showed ICG lymph nodes as revealed by conventional incision. Body Mass Index (BMI) is negatively correlated with lymphatic vessels, and injection depth and fat thickness are the main factors affecting ICG sensitivity (21). However, no correlation was found between lymphatic vessels and the detection rate of fluorescent lymph nodes. In the three cases without lymphatic vessel images, fluorescent lymph nodes were observed through conventional incision. The surgeon’s intuitive feeling is that the difficulty of SLNB is relatively high for obese patients. Hence, the difficulty of surgery must be quantified afterward, and the operation time should be measured.

In this study, the number of ICG lymph nodes was negatively and weakly correlated with BMI. In a similar study, patients with high BMI (≥22 Kg/m²) have fewer removed SLNs than those with lower BMI, but no statistical difference was observed (22).

The advantage of the LFIS over the Langfang imager, devices from Hamamatsu Photonics and Novadaq is its in-situ projection navigation, while obtaining information on lymph node development and adjacent anatomy in the operative field. At the same time, the instrument cost is lower than the previous NIR fluorescence system, including the Langfang imager, devices from Hamamatsu Photonics and Novadaq. One weakness of the LFIs used in the study was that they projected light on the skin at a lower contrast than the screen-developed images of the three devices mentioned earlier, which could cause tiny details to be lost. To solve this problem, we can turn off the operating light during the operation, or increase the brightness of the projection in a subsequent product update.

About the use of near-infrared fluorescence in breast cancer and not just sentinel lymph node biopsy. The Langer’s axillary arch (LAA) is a ventral extension of the anterior margin of the latissimus dorsi. Some breast cancer patients have this mutation, resulting in the failure of a posterior LAA lymph node biopsy or lymph node dissection (23). The LFIS may have great prospects for detecting and managing LAA axillary lymph nodes. Considering the tissue penetration of ICG fluorescence imaging, it is possible to detect the fluorescence signal of lymph nodes behind the LAA without truncation. Compared to radioisotope, ICG has a visual advantage, as the in-situ fluorescence signal of our new technology and the close combination of anatomical structures in the surgical field give surgeons a clearer navigation. If possible, we would like to carry out related studies.

This study has some limitations. First, in this study, ICG fluorescence assisted SLNB was not compared with gold standard method. Secondly, relatively small sample size cannot fully reflect the influencing factors of sentinel lymph node biopsy. For example, we do not have a large enough sample size to determine whether BMI is a factor in the number of sentinel lymph nodes guided by LFIS. But we introduced a new surgical navigation system. The study contributes to the growing evidence of the effectiveness of ICG and provided more opportunities for the use of ICG in SLNB. It is a limitation that this single arm design study also limited our opportunity to compare the quantitative timing of sentinel lymph node biopsies with the two NIR systems.

## Conclusion

The novel real-time *in situ* navigation system is a promising instrument for SLNB in breast cancer. The lymphangiography and SLN development of LFIS are consistent with those of the conventional NIR system. The combination of fluorescence by LFIS and MB may be alternative to the standard method of combined RI and blue dye because of its high detection rate, radiation-free, and operation fluency. This technique satisfies the surgeons’ demand of navigation operation and can be used in various surgical fields.

## Data Availability Statement

The raw data supporting the conclusions of this article will be made available by the authors, without undue reservation.

## Ethics Statement

The studies involving human participants were reviewed and approved by Institutional Research Ethics Committee of the First Affiliated Hospital of Anhui Medical University (PJ2017-11-04). The patients/participants provided their written informed consent to participate in this study.

## Author Contributions

ZW, XY, and PL designed the experiment. ZW and JP analyzed the patient data and wrote papers. CH and YP recorded data. ZW, XY, and JW provided important information of writing and revising manuscript. All authors contributed to the article and approved the submitted version.

## Funding

Anhui Province Science and Technology Major Project (No. 17030801004) and Natural Foundation of Anhui Province (No.2008085MH295).

## Conflict of Interest

The authors declare that the research was conducted in the absence of any commercial or financial relationships that could be construed as a potential conflict of interest.

## Supplementary Material

Image 1 Flow diagram for the progress of patients.

## Abbreviations

NIR system, near-infrared fluorescence imaging systems; SLNB, sentinel lymph node biopsy; MB, methylene blue; RI, Radioisotope; BMI, Body Mass Index; FNR, false negative rate; LAA, Langer’s axillary arch.
