# Supplementary figures and images for: Real-Time In Situ Navigation System With Indocyanine Green Fluorescence for Sentinel Lymph Node Biopsy in Patients With Breast Cancer
Source: Front Oncol. 2021 May 5;11:621914. doi: 10.3389/fonc.2021.621914 (PMC8133435; doi:10.3389/fonc.2021.621914)

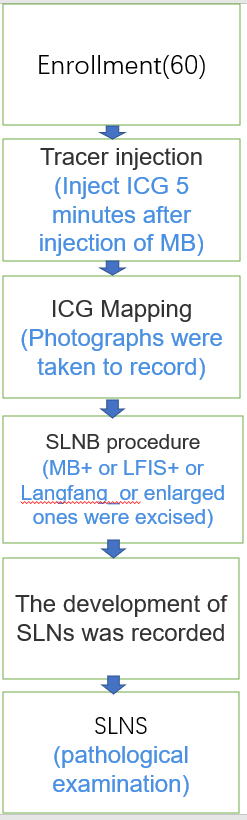

Supplement: Image 1 — Flow diagram for the progress of patients. [file Image_1.png]
